# pH guided pathways trigger tailoring of chiral luminescence in enantiomeric gold cluster assemblies

**DOI:** 10.1039/d5sc04763c

**Published:** 2025-09-10

**Authors:** Camelia Dutta, Jatish Kumar

**Affiliations:** a Department of Chemistry, Indian Institute of Science Education and Research (IISER) Tirupati Tirupati – 517619 Andhra Pradesh India jatish@iisertirupati.ac.in

## Abstract

The modulation of chirality has been a subject of scientific research in past decades due to its broad and impactful applications in drug discovery, catalysis, and biotechnology. However, the control of chirality at the nanoscale is challenging and has recently attracted significant attention due to its implications in diverse fields ranging from medicine to catalysis, optoelectronic devices and materials science. While the ground-state chiral modulation has been a topic of investigation in various nanosystems, the manipulation of excited-state chirality remains relatively underexplored. Herein, we report a straightforward approach to modulate and enhance both the ground and excited state chiral anisotropy in a pair of gold clusters, *via* pH modulations. Gold cluster aggregates synthesized using cysteine as a surface ligand facilitated the adoption of distinct structural features at different pH ranges. By systematically varying the pH across the acidic, isoelectric, and basic regions of the amino acid, the relative engagement of hydrogen bonding and two-point local dipolar interactions could be effectively altered, thereby modulating the nature of cluster aggregation and the resultant chiroptical responses. The pH driven aggregation induced enhanced emission coupled with generation and modulation of cluster assemblies exhibiting the highest among the reported chiral anisotropies for monometallic clusters emphasizes both the novelty of the approach and the potential of the nanomaterials for application in chiral light emitting devices. Moreover, the work provides fundamental insights into the mechanism of pH-dependent supramolecular assembly of nanomaterials.

## Abstract 2

Cysteine-protected gold cluster aggregates with mirror-image CD and CPL were synthesized. pH modulation aided competitive noncovalent interactions causing amplified chiral inversion and aggregation induced emission enhancement.

## Graphical abstract

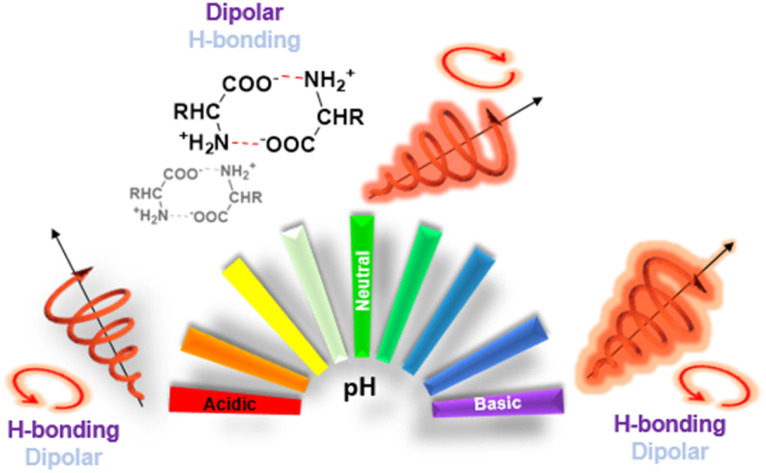

## Introduction

Chirality is an intrinsic property of incoherence that manifests across various length scales in nature, from the subatomic particles to cosmic scales.^1–5^ Although molecular chirality is implicit and widely studied, an understanding of the optical activity at the nanoscale is still in its nascent stages.^6^ Introducing chirality at the nanoscale is particularly challenging, as slight variations in the number or atomic arrangement can significantly influence the optical properties of nanomaterials.^7–9^ Consequently, nanoscale chirality is a blooming area wherein researchers worldwide are actively involved in uncovering the underlying mechanisms and potential applications of chiral nanomaterials.^10–12^ In this context, a wide range of nanomaterials, such as plasmonic nanostructures, quantum dots, carbon-based materials and metal clusters have been actively explored.^13–24^ Among these, metal clusters have recently emerged into the limelight due to their unique size-dependent properties.^25–27^ As the smallest members of the nanomaterial family, metal clusters exhibit the quantum confinement effect, leading to photoemissive properties that open avenues for excited state investigations.^28,29^ Integrating chirality into these emissive clusters opens the door to the intriguing phenomenon of circularly polarized luminescence (CPL), an area that is actively pursued due to its vast potential applications in the field of chiral light emitting devices, chiral sensing and spintronics.^30–36^ The unique properties of CPL active clusters render them compelling materials for further investigation in both fundamental research and practical applications.^37–39^ Various methodologies have been employed to induce chirality in these ultrasmall nanosystems; however, a major bottleneck remains the extremely low efficiency of chiral emission, with anisotropy factors typically in the range of 10^−4^ to 10^−3^.^40–42^ Low emission quantum yield and limited tunability in chiral luminescence have posed major challenges in the use of these materials for practical applications. Therefore, the current state of research calls for innovative strategies to significantly enhance the efficiency of chiral emission with tunable properties. In our very recent work, we addressed this issue by employing both chiral and achiral additives during the cluster synthesis, which triggered the formation of *in situ* cluster aggregates with exceptionally enhanced optical activity and aggregation induced enhanced emission (AIEE).^43^ Herein, we introduce a pH triggered approach for the controlled assembly of clusters leading to aggregated structures exhibiting enhanced and tunable chiral anisotropy. The concept developed herein offers the potential to be applicable to a broad framework of materials, offering a generalizable approach to achieve high *g*_lum_ in various nanosystems.

**Fig. 1:**
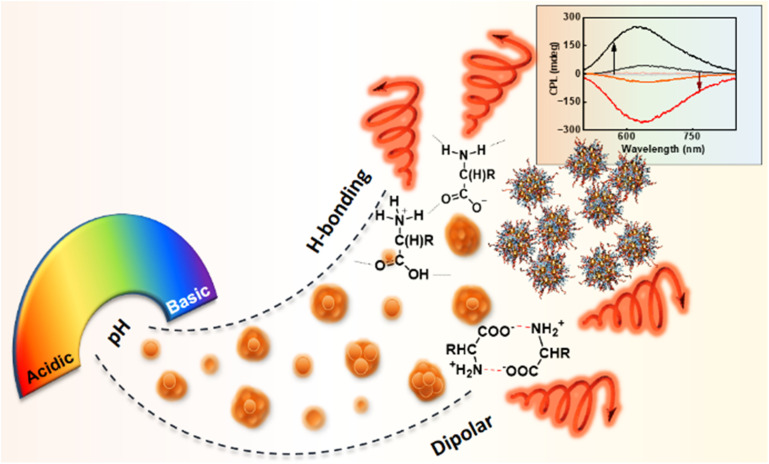
Scheme representing the pH-responsive formation of Au cluster aggregates exhibiting inversion and modulation of chiral luminescence, guided by competitive non-covalent interactions.

Self-aggregated cysteine protected gold (Au) clusters were synthesized adopting a previously reported procedure with modifications.^33^ By precisely adjusting the pH of the medium, both photoluminescence and chiroptical properties of the cluster aggregates could be fine-tuned. The modulation of chiroptical activity was primarily governed by the interplay of two major non-covalent interactions: hydrogen bonding and two-point dipole induced electrostatic interaction. Cysteine, serving as the capping ligand, contains both amine (NH_2_) and carboxyl (COOH) functional groups and exists in three distinct forms at acidic, basic, and isoelectric pH. At an acidic and basic pH, hydrogen bonding dominated the aggregation behaviour, while at the isoelectric point, two-point electrostatic interactions between the ligands played a central role leading to the formation of aggregates having distinct chiral orientation. Hence, through a simple pH modulation strategy, we could successfully achieve AIEE alongside significant control over chiral emission, including its amplification and inversion (Fig. 1). This work demonstrates the highest among the ground and excited state chiral anisotropy reported for monometallic clusters, further highlighting the novelty of the proposed approach.

## Results and discussion

**Fig. 2:**
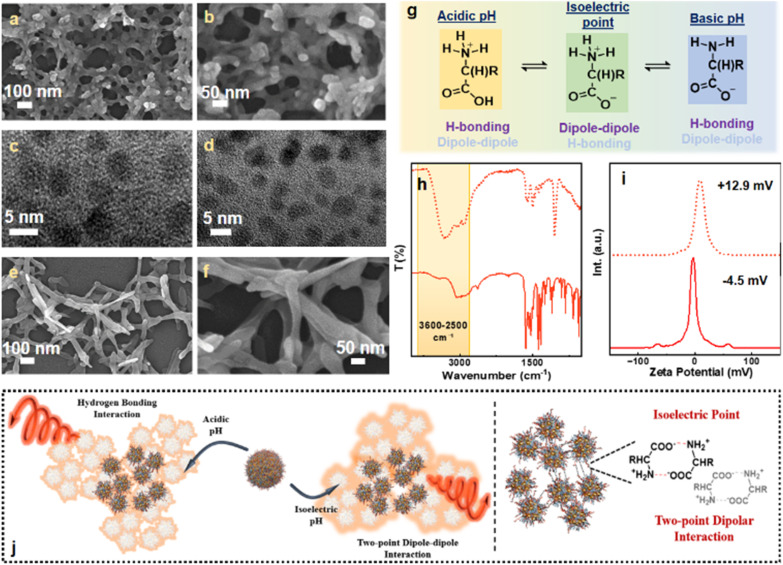
(a, b, e and f) SEM and (c and d) TEM images of the Au clusters synthesized in (a–c) the absence and (d–f) presence of base. (g) Scheme illustrating the molecular structure of an amino acid at different pH. (h) FTIR and (i) zeta potential plots of the clusters synthesized in the absence (dotted trace) and presence (solid trace) of base. (j) Scheme representing the dominating non-covalent forces present at different pH. The right panel represents the two-point dipole moment guided electrostatic interactions responsible for cluster aggregation at the isoelectric pH.

Au clusters were synthesized using cysteine as a capping ligand through the well-established gold–thiol (Au–S) interactions. Interestingly, scanning electron microscopy (SEM) revealed the formation of self-aggregated structures, characterized by interconnected, cross-linked networks featuring distinct nodular structures (Fig. 2a and b). Transmission electron microscopy (TEM) analysis confirmed the presence of individual clusters with an average size of 2.8 nm within the aggregates (Fig. 2c). High-resolution TEM images revealed the presence of lattice planes within the clusters confirming their crystalline nature (Fig. S1). To further investigate the nature of ligand binding on the Au surface, zeta potential measurements were performed in buffer solutions at varying pH. A clear trend in surface charge was observed, with a gradual shift from +15.8 mV to −30.5 mV as the pH increased from acidic (pH = 1) to basic (pH = 13) (Fig. S2). Remarkably, a neutral surface charge was recorded at a pH equivalent to the isoelectric point of cysteine, indicating minimal net charge on the clusters. This result implies the presence of free carboxyl and amine groups on the cluster surface, consistent with the zwitterionic nature of cysteine at the isoelectric point, whereas a protonated amine group and a deprotonated carboxyl group are present at acidic and basic pH, respectively. FTIR spectra indicated the presence of intermolecular hydrogen bonding at both acidic and basic pH, whereas such interactions were absent at the isoelectric point (Fig. S3).^44^ Hence, FTIR data further supported the presence of non-bonded COOH and NH_2_ groups. The absence of –SH stretching confirmed the cysteine interaction with the cluster surface through Au–S bonding.

To gain a deeper insight into the potential of the synthesized clusters towards self-aggregation, zeta potential and FTIR measurements were performed on the cluster solution. Zeta potential analysis of the aggregates indicated a positive surface charge of +12.9 mV (dotted trace, Fig. 2i), while pH measurements confirmed acidic nature with a measured pH of 4.2. These findings suggest the presence of protonated amine (–NH_3_^+^) and carboxyl (COOH) groups on the cluster surface. The existence of the COOH groups is indicative of their potential to engage in intermolecular hydrogen bonding, which is likely responsible for the self-assembly and aggregation of the clusters. This hypothesis was further substantiated by FTIR measurements revealing a blue-shifted and intensified stretching band in the 3600–2500 cm^−1^ region, characteristic of hydrogen-bonding interactions (dotted trace, Fig. 2h). Collectively, these results support the hypothesis that pH-dependent hydrogen bonding plays a critical role in driving the aggregation behaviour in cysteine-capped Au clusters at acidic pH.

We next aimed to investigate the role of the isoelectric point of cysteine in governing the interaction and aggregation behaviour of the Au clusters. At the isoelectric point, cysteine molecules are expected to adopt a zwitterionic form, possessing both protonated amine (–NH_3_^+^) and deprotonated carboxyl (–COO–) groups (Fig. 2g). Under these conditions, the cysteine moieties are anticipated to possess an overall neutral charge. This zwitterionic state introduces two opposite dipolar centers at two ends of the molecule, promoting two-point complementary dipolar interactions among the cysteine units, leading the individual cluster molecules to approach each other.^45,46^ Consequently, rather than hydrogen bonding, dipolar interactions between oppositely charged functional groups on neighbouring molecules are also anticipated, potentially facilitating cluster assembly yielding different morphologies. To explore this, the pH of the synthesis medium was adjusted to 5.9, slightly above the isoelectric point of cysteine, by adding a base during the synthesis. SEM analysis revealed the presence of aggregated structures composed of clusters forming network-like architectures with tubular termini, in contrast to the nodular features observed in acidic medium (*vide supra*). This morphological shift suggests the predominance of a different interaction mechanism governing the assembly process under these conditions (Fig. 2e and f). TEM images confirmed that these aggregates were composed of discrete, individual clusters (Fig. 2d). FTIR spectroscopy indicated a weakening of hydrogen bonding interactions under these conditions, as evidenced by reduced intensity and shifting of the characteristic stretching bands in the 3600–2500 cm^−1^ region (solid trace, Fig. 2h). This observation suggests that at the isoelectric point, hydrogen bonding alone is insufficient to drive the aggregation process. Instead, the molecules adopt a dipolar nature promoting dipole moment driven two-point electrostatic interactions as the primary contributor to the aggregation process.^46^ The self-assembled structures exhibiting distinct morphologies highlights the role of surface states in significantly influencing the non-covalent interactions. Zeta potential measurements showed a low negative surface charge of −4.5 mV, in agreement with the presence of a small proportion of deprotonated carboxylate groups (–COO^−^) at a pH slightly above the isoelectric point. These findings underscore the ability to tune aggregation pathways through precise pH control thereby regulating the interparticle interactions and, in turn, the morphology of Au cluster aggregates (Fig. 2j).

**Fig. 3:**
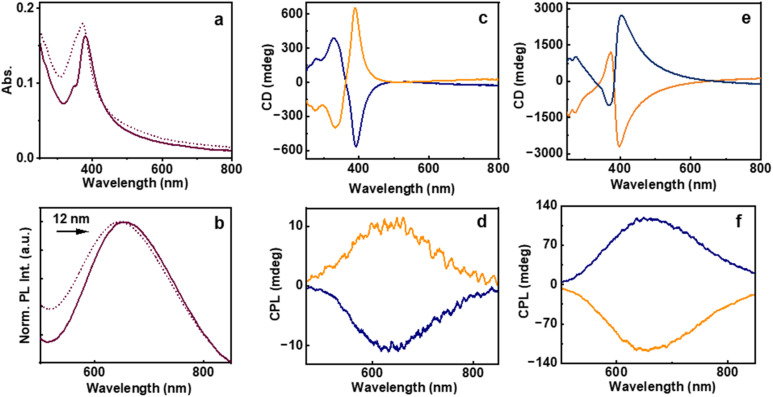
(a) UV-vis and (b) PL spectra of the Au clusters synthesized at pH 4.2 (dotted trace) and 5.9 (solid trace). (c and e) CD and (d and f) CPL spectra of the Au clusters synthesized in the (c and d) absence of base (pH = 4.2) and (e and f) the inverted spectra upon *in situ* base addition (pH = 5.9). Blue and orange traces correspond to the clusters synthesized using l- and d-cysteine, respectively.

The photophysical properties of the synthesized clusters were systematically investigated using UV-visible absorption (UV-vis) and photoluminescence (PL) spectroscopy. The UV-vis spectra demonstrated a broad absorption band extending across both the ultraviolet and visible regions of the electromagnetic spectrum, with a distinct absorption maximum centered around 370 nm (dotted trace, Fig. 3a). The broad absorption profile arising due to enhanced light scattering further supports the formation of aggregates. PL excitation spectra exhibited a relatively sharper peak further highlighting the influence of excess photon scattering leading to the broad absorption profile (Fig. S4). A comparable absorption profile was observed for the aggregates formed at pH 5.9, although a red shift of 11 nm was evident, indicating changes in the electronic levels upon aggregation. A distinct shoulder peak observed around 345 nm at this pH is attributed to extended non-bonded Au–Au interactions, likely arising from electronic transitions involving the promotion of an electron from the filled 5d_*z*^2^_ orbital to the vacant 6p_*z*_ orbital.^47^ Hence, as the Au cluster molecules approach each other by dipolar interactions, aurophilic interactions may significantly contribute to the aggregation mechanism. These weak, non-bonded, attractive forces between Au(i) centers are known to facilitate the assembly of Au(i) complexes, thereby playing a potentially crucial role in the clustering behaviour. The aggregates in acidic medium exhibited PL with an emission maximum at 644 nm. However, the clusters prepared at pH 5.9 showed a red shift of 12 nm in the emission peak, accompanied by a significant enhancement in fluorescence intensity (Fig. 3b). This was reflected by an increase in the quantum yield from 7.87% to 11.94%, suggesting improved radiative recombination efficiency, as a result of AIEE. The observed AIEE further supports the formation of distinct morphological aggregates at an elevated pH which is consistent with the SEM images (*vide supra*).^33^ Under acidic conditions, the excited-state lifetimes of the clusters obtained using the multichannel scaling (MCS) mode exhibited a biexponential decay profile for both the enantiomers, suggesting the presence of heterogeneous emissive states. d-Cysteine protected Au clusters exhibited lifetimes of 0.81 μs (46.31%) and 3.0 μs (53.69%), resulting in an average lifetime of 1.99 μs; whereas for the opposite isomer, lifetimes were calculated to be 1.0 μs (52.87%) and 3.86 μs (47.13%) with an average value of 2.35 μs (Fig. S5). At the isoelectric pH, average values of 3.02 μs and 4.03 μs were observed for the l- and d-cysteine-derived clusters, respectively, indicating that the pH variation resulted in an enhancement in the excited state lifetime (Fig. S6), supportive of the enhanced aggregation.

The incorporation of optically active molecules as surface-passivating ligands during the synthesis prompted a systematic investigation into their chiroptical properties. Given the intrinsic chirality of these ligands, it was hypothesized that their spatial arrangement around the metallic core could induce optical activity in the resulting nanostructures. To explore these effects, circular dichroism (CD) and circularly polarized luminescence (CPL) measurements were carried out. The CD spectra of the cluster aggregates synthesized at acidic pH with opposite enantiomers of cysteine displayed mirror-image profiles, confirming the enantiospecific transfer of chirality from the ligand to the cluster assembly (Fig. 3c). This observation provides strong evidence for the chiral nature of the supramolecular organization within the aggregates. The CD spectra showed a zero-crossover feature that coincided with the excitation maximum of the cluster aggregates. This observation underscores the significant influence of the ligand chirality on modulating both the photophysical and chiroptical behaviour as well as the supramolecular architectures of the cluster assemblies. The aggregates exhibited a chiral absorption dissymmetry (*g*_abs_) of −0.015 and +0.019 for the clusters synthesized using l- and d-cysteine, respectively (Fig. S7a). The pronounced PL and strong CD signals prompted further investigation into their excited state chirality. CPL measurements yielded a clear mirror-image profile for aggregates synthesized using enantiomeric d- and l-cysteine ligands, consistent with the corresponding sign of CD spectra (Fig. 3d). The aggregates prepared using l-cysteine exhibited a *g*_lum_ value of −0.005, whereas those synthesized using d-cysteine showed the corresponding value of +0.003 (Fig. S7b). These findings illustrate the effective induction of chirality from the ligand framework to the electronic transitions of the cluster aggregates, highlighting the critical role of ligand stereochemistry in dictating the chiroptical properties of the clusters in their excited state.

The chiroptical behaviour of the cluster aggregates synthesized at a pH 5.9 was particularly noteworthy. At this specific pH, both CD and CPL spectra exhibited an inversion and broadening of the signal relative to the aggregates formed under acidic pH (Fig. 3e and f). This reversal suggests a significant modification in the chiral organization of the clusters. The underlying cause of inversion is attributed to changes in hydrogen bonding interactions within the aggregates.^48–51^ In alignment with the observed inversion of chiroptical signals, both the *g*_abs_ and *g*_lum_ exhibited notable enhancement at pH close to the isoelectric point. For the l- and d-cysteine derived aggregates, *g*_abs_ values of +0.055 and −0.039 were observed, respectively, whereas the corresponding *g*_lum_ values were found to be +0.017 and −0.015, respectively (Fig. S8). The formation or disruption of hydrogen bonds during supramolecular assembly of molecules is known to induce a reconfiguration of the chiral framework, resulting in an opposite optical rotation and emission polarization. Such reversal in chiral luminescence arising from the modulation of molecular interactions was recently reported by our group and others.^43,50–53^ These results highlight the critical influence of hydrogen bonding on the chiral structural arrangement and the associated optical properties of the cluster assemblies.

Both the aggregate systems exhibited exceptional structural stability; a characteristic benefit commonly associated with *in situ* formed assemblies. Notably, their chiroptical signatures, as evidenced by CD spectroscopy, remained unaltered even after extended exposure to high-intensity ultrasonication (Fig. S9).

This resilience suggests the presence of robust non-covalent interactions maintaining the integrity of the supramolecular architecture. Given that thermal stability is a critical parameter for the practical deployment of functional materials, we further investigated the temperature-dependent behaviour of the aggregates. The CD spectra were recorded while incrementally heating the samples from 10 °C up to 90 °C, followed by cooling back to ambient conditions. The absence of any significant spectral deviations during the thermal cycle confirmed that the aggregates retain their chiral organization and structural orientation even under elevated thermal stress (Fig. S10 and S11). Collectively, these findings affirm the high mechanical and thermal stability of the aggregates, reinforcing their suitability for application in demanding environments.

To further explore the mechanism behind the altered chiroptical properties of the aggregates in the presence of NaOH, a control experiment was performed using KOH under identical synthetic conditions. This experiment was aimed to assess the specific role of sodium (Na^+^) ions in influencing the aggregation behaviour and resulting chiral optical activity. The aggregates formed in the presence of KOH exhibited chiroptical signals comparable to those observed with NaOH (Fig. S12). The consistency across both conditions effectively rules out any direct role of Na^+^ in modulating the chiral assembly. Instead, the findings strongly suggest that the changes in pH, rather than the counter alkali metal cation, are responsible for the observed variations in aggregation pattern and the chiroptical activity. Hence, the increase in pH affects the intermolecular interactions guiding the self-assembly, leading to the observed structural and optical changes.

**Fig. 4:**
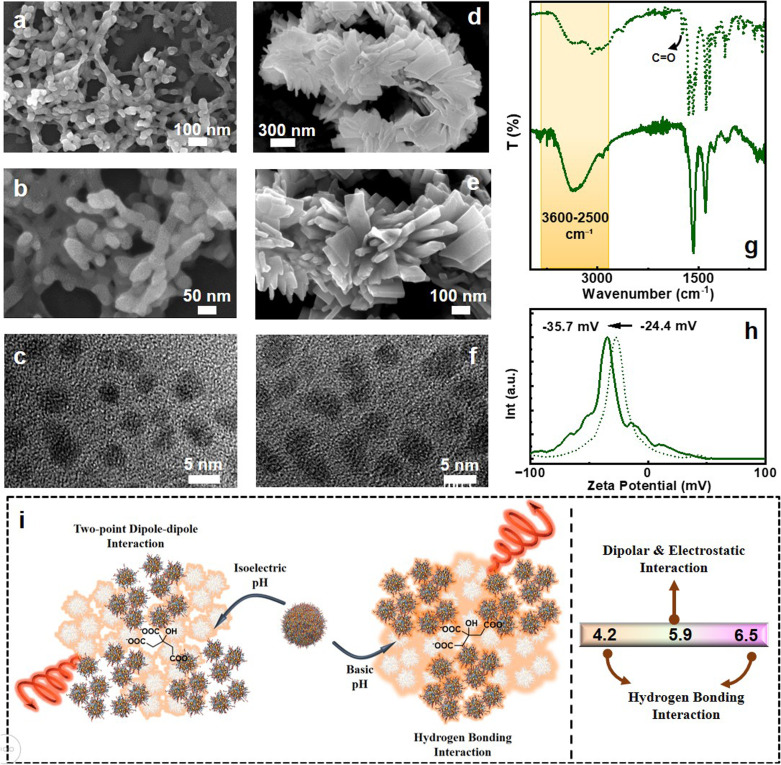
(a, b, d and e) SEM and (c and f) TEM images of the citrate mediated Au cluster aggregates synthesized at a pH of (a–c) 5.9 and (d–f) 6.5. (g) FTIR spectra and (h) zeta potential plots of the clusters synthesized at pH 5.9 (dotted trace) and 6.5 (solid trace). (i) Scheme exhibiting the dominating forces under both the pH conditions.

Following our successful investigation into the effects of acidic and isoelectric pH on the interactions between individual clusters, we aimed to explore the influence of basic conditions. To this end, we initially increased the base concentration during synthesis; however, the reaction failed to proceed under strongly basic conditions. To address this limitation, sodium citrate was employed as a mild reducing agent to perform citrate-mediated synthesis of Au clusters. In addition to its reducing properties, the negative charge on citrate is anticipated to promote electrostatic interactions with the Au clusters, driving cluster aggregation primarily through the charge complementarity. The resulting clusters exhibited a pH of 5.9. SEM analysis again confirmed the formation of aggregated structures with a distinct morphology (Fig. 4a and b). Consistent with our earlier findings, the pH value, being closer to the isoelectric point, suggests non-hydrogen bonding charge complementary interaction to be the dominant force responsible for cluster assembly, which was further expected to be promoted by citrate induced electrostatic interaction and the Au(i)–Au(i) aurophilic interaction. TEM imaging revealed individual Au clusters distributed within the aggregates (Fig. 4c). To further elevate the pH, we repeated the citrate-mediated synthesis in the presence of an optimized base concentration. This resulted in Au clusters with a pH of 6.5, clearly above the isoelectric point, thus providing a system to investigate the behaviour of cluster interactions under relatively basic conditions. SEM analysis revealed a clear shift in morphology to the plate-like structures, consistent with the presence of individual clusters within the aggregates as confirmed by TEM imaging (Fig. 4d–f).

The observation of aggregate formation at the same pH of 5.9 but with distinct morphologies, ranging from tubular to nodular networks depending on the presence of citrate, highlights the significant impact of the chemical environment on both self-assembly and co-assembly of clusters. Citrate, due to its multidentate nature and multiple negatively charged carboxylate groups, can interact with the surface ligands comprising NH_3_^+^, thereby influencing the local coordination environment and ionic strength. These interactions can modulate the relative contributions of various non-covalent forces. Contrarily, the presence of base (pH = 6.5) alters the dominant interaction pathways from electrostatic to hydrogen bonding interaction, leading to distinct aggregation behaviours resulting in different morphological outcomes.

To further investigate the underlying interaction mechanisms, FTIR analysis was performed on both the citrate-mediated Au cluster systems. As expected, the sample with a pH of 5.9, close to the isoelectric point, did not display spectral signatures associated with hydrogen bonding (dotted trace, Fig. 4g). In contrast, the sample prepared under relatively basic conditions exhibited a pronounced and broadened vibration band in the 3600–2500 cm^−1^ region, indicative of hydrogen bonding (solid trace, Fig. 4g). The shift and increased intensity of these stretching frequencies confirm the formation of hydrogen-bonded interactions at an elevated pH due to the presence of free COOH. These results are consistent with previous findings: near the isoelectric point, two-point electrostatic interactions dominate due to the relatively balanced distribution of positive and negative functional groups within the protecting ligands; however, at a higher pH, hydrogen bonding is the dominant force as electrostatic interaction is unfavourable due to the increased abundance of anionic functional ends (Fig. 4i). In addition to these, the presence of negatively charges citrate ions provides additional pathways for electrostatic interactions. Zeta potential measurements provide further validation of these findings, with both citrate-mediated systems exhibiting negative values of −24.4 mV (pH = 5.9) and −35.46 mV (pH = 6.5) (Fig. 4h). At a pH closer to the isoelectric point (pH = 5.9), where the net surface charge would typically approach neutrality, the presence of anionic citrate contributes to measurable negative surface potential instead of a value close to zero. This indicates active interactions between the citrate ions and the surface of the colloids, suggesting a dynamic modification of surface charge. Under basic conditions, the zeta potential achieves even higher negative value, which can be attributed to a decrease in NH_3_^+^ and a corresponding increased abundance of COO^−^. These results highlight the combined influence of pH and citrate coordination on colloidal surface chemistry.

**Fig. 5:**
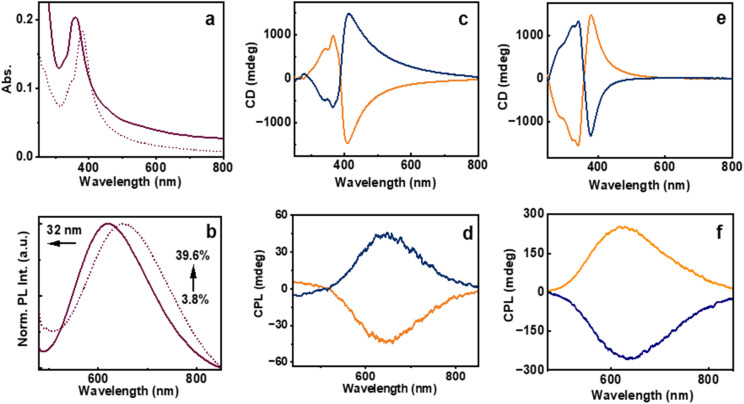
Spectral properties of the cluster aggregates synthesized in the presence of citrate: (a) UV-vis and (b) emission spectra of the citrate mediated Au clusters synthesized in the absence (dotted trace) and presence (solid trace) of base. (c and e) CD and (d and f) CPL spectra of the clusters synthesized (c and d) in the absence of base (pH = 5.9) and (e and f) inverted spectra upon synthesis in the presence of base (pH = 6.5). Blue and orange traces correspond to the clusters synthesized using l- and d-cysteine, respectively.

Of particular interest were the photophysical and chiroptical properties of the citrate-mediated Au cluster aggregates. Both citrate-based samples, synthesized with and without the addition of a base, demonstrated similar absorption and emission profiles. At a pH of 5.9, a shoulder peak in the absorption profile emerged at 345 nm supporting the Au(i)–Au(i) aurophilic interaction (Fig. 5a). A pronounced enhancement in PL quantum yield was observed for the sample prepared under relatively basic condition. The sample synthesized in the presence of base showed an increased PLQY of 39.6%; a 10.4-fold enhancement as compared to the sample synthesized at a pH of 5.9 with a PLQY of 3.8% (Fig. 5b). Despite the overall similarity in spectral characteristics, blue shifts of 18 nm and 32 nm in the absorption and emission maximum, respectively, for the base-assisted sample suggested minor modifications in the electronic structure upon variation in the order of aggregation (Fig. 5a and b). These results suggest that incorporating a base during synthesis markedly affects the photophysical properties of the clusters, potentially by adjusting the pH of the medium, which in turn influences the interparticle interactions. The citrate-stabilized Au clusters synthesized with l- and d-cysteine exhibited excited-state lifetimes comparable with the citrate free pair of Au clusters. l-Cysteine stabilized Au clusters exhibited an average lifetime 1.87 μs whereas the opposite isomer exhibited an average value of 2.47 μs (Fig. S13). However, when the synthesis was conducted in a basic environment at pH 6.5, the average lifetimes of the l- and d-Au clusters shifted to 2.20 μs and 2.19 μs, respectively (Fig. S14).

Particularly noteworthy were the chiroptical features of the citrate-stabilized Au clusters. At pH 5.9, the citrate-mediated Au clusters exhibited CD and CPL signals with the same sign as those observed in citrate-free Au clusters having the same pH, suggesting analogous chiral configurations guided by similar dominant non-covalent forces in both the systems (Fig. 5c and d). The *g*_abs_ values at pH 5.9 were calculated to be +0.066 and −0.079 for the citrate mediated clusters protected with l- and d-cysteine, respectively, whereas, the corresponding *g*_lum_ values were found to be +0.012 and −0.015 (Fig. S15). The chiroptical features appear to be primarily governed by the dipolar-electrostatic interactions over hydrogen bonding interactions, both (i) among the clusters and (ii) between the clusters and the surrounding citrate anions. Interestingly, upon increasing the pH to 6.5 by the incorporation of a base *in situ*, clusters displayed an inversion with marked enhancement in both the CD and CPL signals (Fig. 5e and f). A shift in pH from 5.9 to 6.5 resulted in inverted *g*_abs_ values of −0.074 and +0.05 for cluster aggregates stabilized with l- and d-cysteine, respectively (Fig. S16a). The *g*_lum_ values for the same clusters were calculated to be −0.028 and +0.029 (Fig. S16b), which are among the highest chiral dissymmetry values reported for any cluster (Table 1). Such tuning of optical signals indicates a reversal in the dominant chiral structure, aligning the chiroptical signature closely with that of the citrate free Au clusters at pH 4.2. Such behaviour underscores the sensitivity of the system's chiral properties to pH changes driven by non-covalent interactions. Hence, at pH values far from the isoelectric point, hydrogen bonding interactions were dominant, resulting in chiroptical activity with consistent chiral handedness. In contrast, near the isoelectric point, dipolar interactions prevailed, leading to an inversion in the chiroptical response. Though the *g*_abs_ and *g*_lum_ values in all these cases might have a contribution from the scattering effect, the excitation spectral data (Fig. S4) as well as the consistency in handedness between ground and excited chiroptical data render this effect negligible in these systems.

**Table 1:** Summary of the *g*_abs_ and *g*_lum_ values of Au cluster aggregates synthesized at varying pH

	pH	l-Cysteine		d-Cysteine	
		*g*_abs_	*g*_lum_	*g*_abs_	*g*_lum_
Au cluster aggregate	4.2	−0.015	−0.005	+0.019	+0.003
	5.9	+0.055	+0.017	−0.039	−0.015
Citrate Au cluster aggregate	5.9	+0.066	+0.012	−0.079	−0.015
	6.5	−0.074	−0.030	+0.05	+0.030

The concentrations of sodium citrate and the base were systematically optimized. Initially, batches of citrate-mediated Au cluster synthesis were carried out to determine the exact amount of citrate necessary to attain a pH of 5.9. Once the optimal citrate concentration was established (Fig. S17), subsequent synthesis was carried out by varying the base concentration to identify the pH at which chiral inversion occurs. This optimization revealed that a minimum pH of 6.5 is critical for inducing the observed CD inversion (Fig. S18). To exclude any potential influence of sodium ions on the observed phenomenon, parallel synthesis was performed using potassium citrate. The resulting CD behaviour remained consistent (Fig. S19), thereby confirming that the inversion is independent of the alkali metal ion and is driven by pH effects. Similar to the previous system, the robustness of the citrate-mediated Au cluster aggregates was thoroughly examined by exposing them to harsh mechanical and thermal stress, including extended ultrasonication and elevated temperatures. Notably, neither prolonged sonication nor thermal exposure resulted in any discernible alteration of the CD signals (Fig. S20–S22). The clusters were re-dissolved under different pH conditions and subjected to photophysical and chiroptical investigations. The spectral features remained unchanged under post-synthetic pH variation (Fig. S23), confirming that aggregation occurs exclusively during synthesis and the resulting structures remain stable over a broad pH range. This outcome demonstrates that the chiral features of the system remained unaffected under extreme environmental conditions, highlighting the structural integrity and durability of the assembled cluster framework.

## Conclusions

In summary, we introduce a straightforward and novel approach for enhancing chiral anisotropy in metal clusters. Enantiomerically pure Au clusters were synthesized, forming distinct aggregates depending on the pH of the medium. The nature of the dominant non-covalent interactions was found to play a crucial role under different pH conditions: hydrogen bonding governed the assembly at both low and high pH, while two-point dipolar interactions were predominant near the isoelectric point of the ligand. This pH-dependent modulation not only affected the aggregation behaviour but also had a pronounced impact on the physico-optical activity of the systems, resulting in AIEE with chiral signal inversion. The first inversion occurred as the pH increased from acidic to the isoelectric point, driven by a transition from hydrogen bonding to two-point dipolar interactions. A second inversion was observed on moving from the isoelectric point to basic pH, wherein hydrogen bonding again was the dominant non-covalent interaction. These findings demonstrate that pH tuning can serve as a powerful tool to control both the structure and chiroptical properties of Au cluster assemblies, with significant enhancements observed in both ground- and excited-state chiral responses. The facile approach demonstrated herein opens avenues for the development of materials that can be used in chiral catalysis, chiral light emitting devices and data storage.

## Author contributions

J. K. conceived and coordinated the project. C. D. carried out the experiments. C. D. & J. K. analysed and consolidated the data. C. D. and J. K. prepared the manuscript. Both authors have given approval to the final version of the manuscript.

## Conflicts of interest

There are no conflicts to declare.

## Supplementary Material

SC-016-D5SC04763C-s001

## Data Availability

The data supporting this article have been included as part of the SI. The SI file contains experimental details on the synthesis of clusters and their pH modulated aggregation, characterization of cluster aggregates using FTIR, zeta potential, PL and lifetime, effect of temperature and sonication. See DOI: https://doi.org/10.1039/d5sc04763c.
