# Supplementary material for: pH guided pathways trigger tailoring of chiral luminescence in enantiomeric gold cluster assemblies
Source: Chem Sci. 2025 Sep 10;16(40):18713–21. doi: 10.1039/d5sc04763c (PMC12439383; doi:10.1039/d5sc04763c)
Supplement: SC-016-D5SC04763C-s001 [file SC-016-D5SC04763C-s001.pdf]

Supplementary Information (SI) for Chemical Science.
This journal is © The Royal Society of Chemistry 2025

Supporting Information

pH Guided Pathways Trigger Tailoring of Chiral Luminescence in Enantiomeric Gold Cluster Assemblies

Camelia Dutta and Jatish Kumar*

Department of Chemistry, Indian Institute of Science Education and Research (IISER) Tirupati, Tirupati – 517619, Andhra Pradesh, India.

Email: jatish@iisertirupati.ac.in

Table of Contents

Sl. No.	Content	Page No.
1	Experimental details	S2-S3
2	Fig. S1: HRTEM images of Au cluster aggregates	S4
3	Fig. S2: Zeta potential plot of the Au cluster aggregates	S4
4	Fig. S3: FTIR spectra of Au cluster aggregates	S5
5	Fig. S4: photoluminescence excitation spectra of the Au clusters	S5
6	Fig. S5: Lifetime decay plot of the Au cluster aggregates at acidic pH	S6
7	Fig. S6: Lifetime decay plot of the Au cluster aggregates at pH 5.9	S6
8	Fig. S7: g_{abs} and g_{lum} plot of the Au cluster aggregates at acidic pH	S6
9	Fig. S8: Chiral anisotropy plot of the Au cluster aggregates at pH 5.9	S7
10	Fig. S9: Effect of sonication on the Au cluster aggregates	S7
11	Fig. S10: Temperature dependent CD under acidic pH	S7
12	Fig. S11: Temperature dependent CD at pH 5.9	S8
13	Fig. S12: Effect of KOH on Au cluster aggregation	S8
14	Fig. S13: Lifetime plots of the citrate Au cluster aggregates at pH 5	S8
15	Fig. S14: Lifetime plots of the citrate Au cluster aggregates at pH 6.5	S9
16	Fig. S15: g_{abs} and g_{lum} plots of citrate Au cluster aggregates at pH 5.9	S9
17	Fig. S16: g_{abs} and g_{lum} plot of citrate Au cluster aggregates at pH 6.5	S9
18	Fig. S17: Optimization of sodium citrate concentration	S10
19	Fig. S18: Optimization of NaOH concentration for chiral inversion	S10
20	Fig. S19: Effect of potassium citrate on Au cluster aggregation	S11
21	Fig. S20: Effect of sonication on citrate Au cluster aggregation	S11
22	Fig. S21: Temperature dependent CD of citrate aggregates at pH 5.9	S11
23	Fig. S22: Temperature dependent CD of citrate aggregates at 6.5 pH	S12
24	Fig. S23: Post-synthetic pH dependent studies	S12

Supporting Information

Experimental Details:

Chemicals and Materials

Gold(III) chloride trihydrate ($HAuCl_4$) was purchased from Sigma-Aldrich. D/L-cysteine hydrochloride monohydrate was purchased from TCI Chemicals. Sodium hydroxide (NaOH), potassium hydroxide (KOH), sodium citrate tribasic and potassium citrate were purchased from SRL chemicals. All the glass wares were washed with aqua regia ($HCl:HNO_3$ = 3:1), milli-Q water, and acetone and were dried in oven at 60 °C. Milli-Q water was consistently utilized throughout the entirety of the study.

Characterization

UV-visible absorption, photoluminescence, and CD spectrum were collected using Cary UV-vis Multicell Peltier, JASCO spectrofluorometer FP-8500, and JASCO J-1500 CD spectrometer, respectively. Zeta potential measurements were conducted using the Litesizer 500. FT-IR spectra was collected by BRUKER ECD-ART instrument. Lifetime measurements were carried out on Edinburg FLS-1000 fluorescence spectrometer, with an excitation laser of 375 nm. Absolute quantum yield measurements were acquired employing the integrating sphere within an Edinburg FLS-1000 instrument. Morphology of the samples were investigated using SEM, FESEM Gemini 560, Germany. TEM images were captured using FEI Tecnai G2 20 S-twin electron microscope with an acceleration voltage of 200 kV..

Synthesis of Au Cluster Aggregates

The synthesis of gold (Au) clusters was carried out by introducing $HAuCl_4$ into an aqueous cysteine solution. In details, the cysteine solution was prepared by adding 63.6 µL of a 569.38 mM D/L-cysteine stock solution to 4.93 mL of milli-Q water, followed by thorough mixing. Subsequently, 2.94 µL of the 2.03 M $HAuCl_4$ stock solution was introduced into the cysteine solution. The reaction mixture was then incubated at room temperature under continuous stirring at 400 rpm under dark. After the continuous stirring of 24 hours, self-organised Au cluster aggregates were formed. The synthesized clusters were purified by dialysis and exhibited a pH of 4.2. The sample was stored at 4 °C for further studies. For photophysical characterization, a 75 µL aliquot of the synthesized clusters was diluted to a final volume of 2 mL. For obtaining the cluster solution with 5.9 pH, 1M 60 µL NaOH has been added into the cysteine solution and stirred for 5 min before the introduction of $HAuCl_4$. The cluster solution was again purified and stored following the same procedure as mentioned above.

Synthesis of Citrate-mediated Au Cluster Aggregates

The synthesis of citrate-mediated Au clusters was carried out by introducing $HAuCl_4$ into an aqueous solution containing cysteine and sodium citrate. In details, the cysteine solution was prepared by adding 63.6 µL of a 569.38 mM D/L-cysteine stock solution to 4.53 mL of milli-Q water. Upon mixing it well, 400 µL of 500 mM sodium citrate solution was introduced followed by thorough mixing for 5 min. Subsequently, 2.94 µL of the 2.03 M $HAuCl_4$ stock solution was introduced into the cysteine solution. The reaction mixture was then incubated at room temperature under continuous stirring at 400 rpm under dark. After the continuous stirring of 24 hours, self-organised Au cluster aggregates were formed at a pH of 5.9. For synthesis of cluster aggregates at pH 6.5, same procedure as described above was followed. However, before the addition of sodium citrate, NaOH was added into the cysteine containing solution to adjust the pH and mixed for 5 min, followed by the citrate and $HAuCl_4$ addition following the same procedure described above.

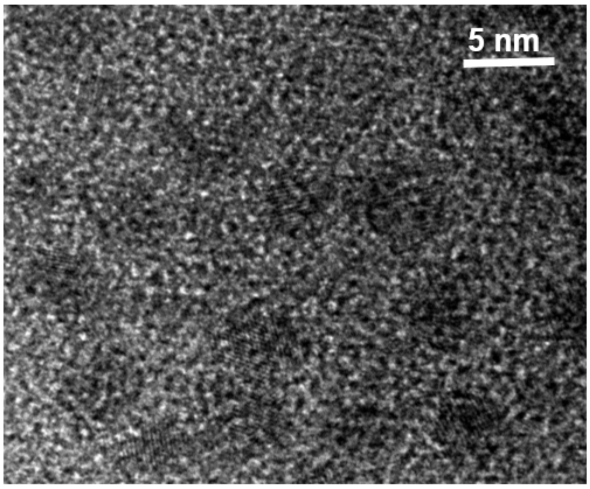

Figure S1. HRTEM Images of the Au clusters showing the crystalline lattice places within each cluster.

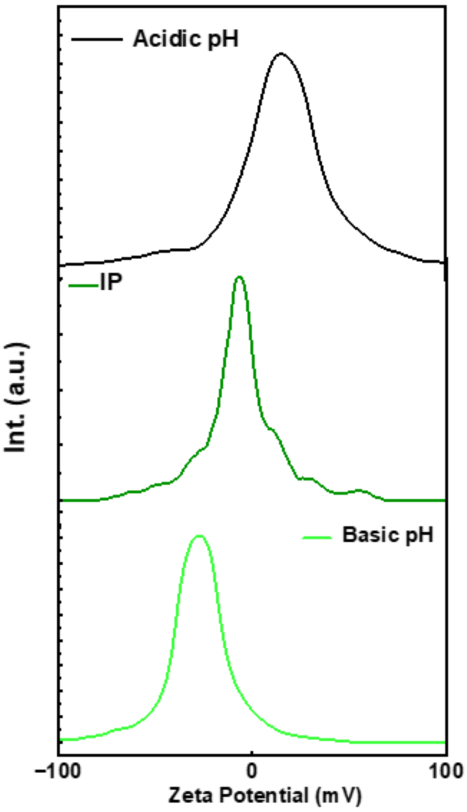

Figure S2. Zeta potential plot of the Au clusters under different pH.

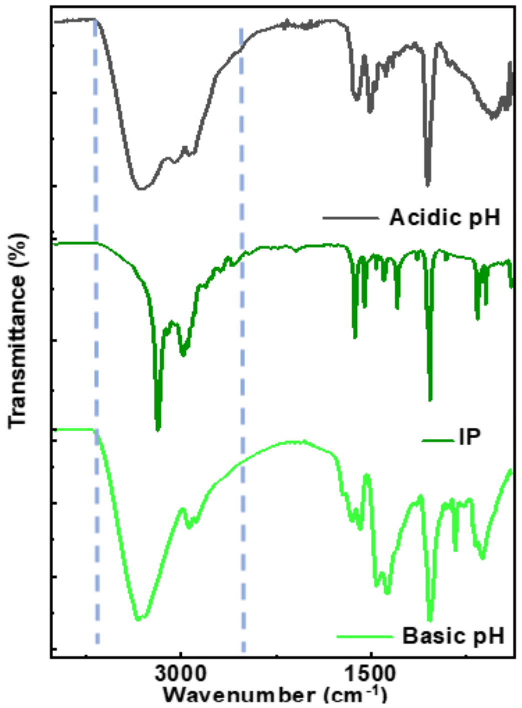

Figure S3. FT-IR spectrum of the Au clusters under different pH.

A significant change in the intermolecular interactions between the clusters was observed when the pH shifted from acidic to basic, as reflected in the spectral features within the 3600–2500 cm^{-1} region. Notably, intermolecular hydrogen bonding was evident under both acidic and basic conditions, but was absent at the zwitterionic pH.

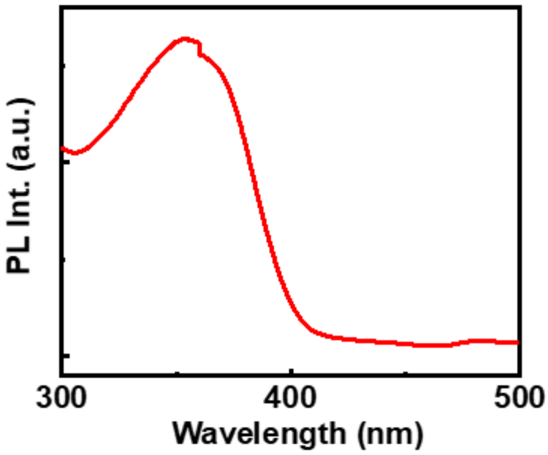

Figure S4. PL excitation spectra of the Au clusters for emission monitored at 350 nm.

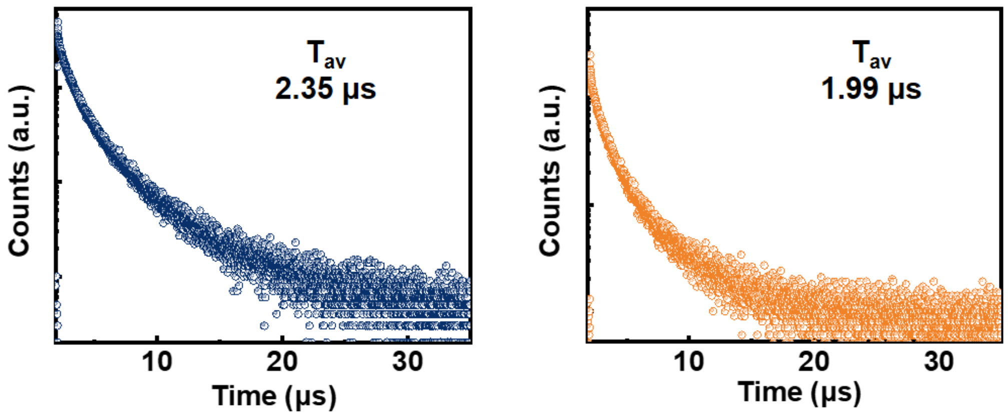

Figure S5. Lifetime decay plot of the Au clusters synthesized at pH 4.2 using L- (blue trace) and D- (orange trace) cysteine.

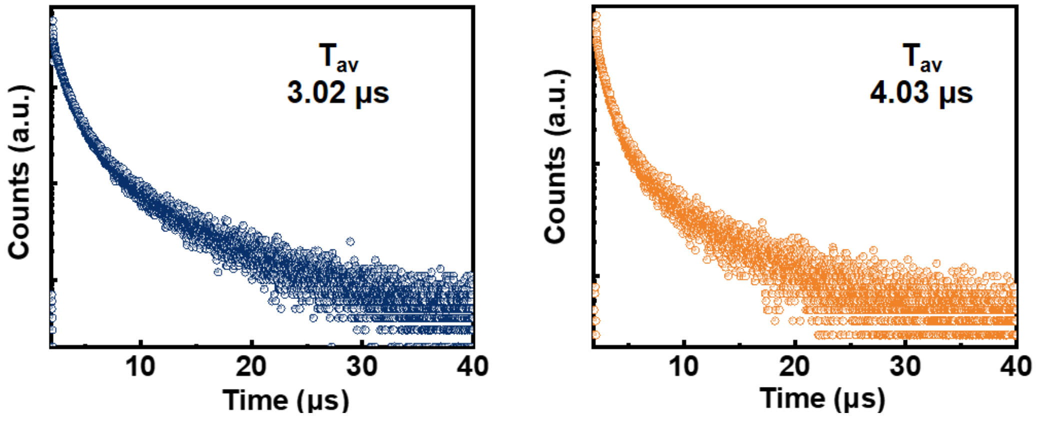

Figure S6. Lifetime decay plot of the Au clusters synthesized at pH 5.9 using L- (blue trace) and D- (orange trace) cysteine.

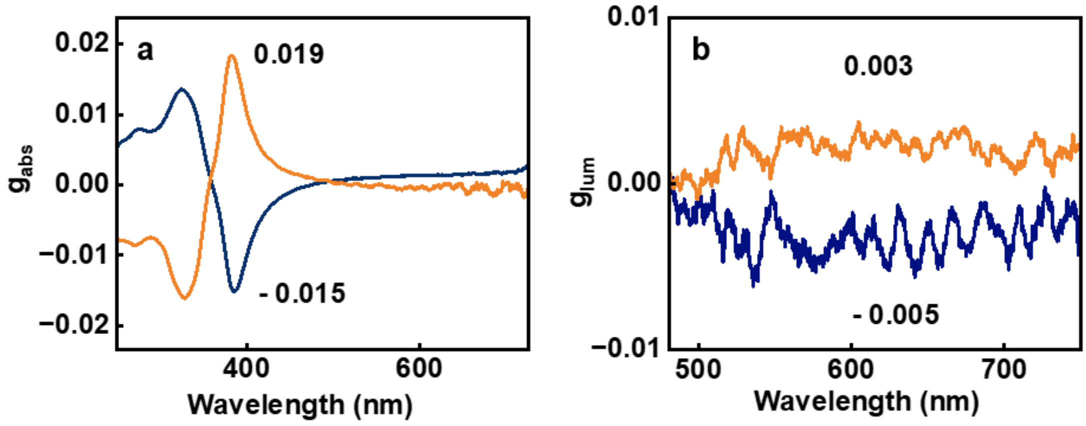

Figure S7. (a) g_{abs} and (b) g_{lum} plot of the Au cluster aggregates synthesized at a pH of 4.2. Blue and orange traces are corresponding to the Au clusters synthesized in presence of L- and D-cysteine, respectively.

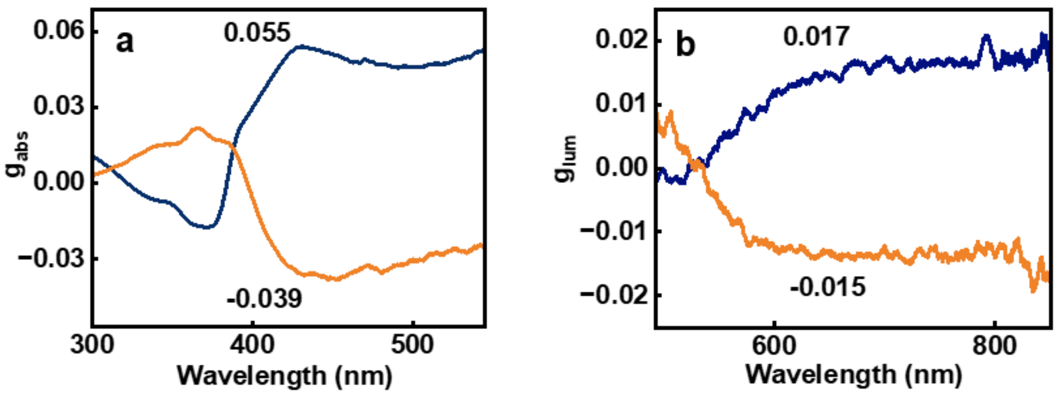

Figure S8. (a) g_{abs} and (b) g_{lum} plot of the Au clusters synthesized in presence of base (pH = 5.9). Blue and orange traces correspond to the Au clusters synthesized in presence of L- and D-cysteine, respectively.

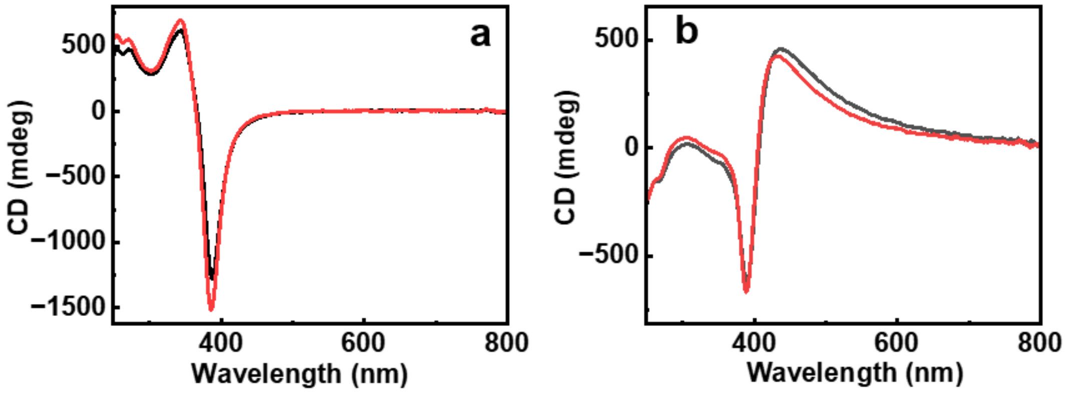

Figure S9. Effect of sonication on the Au cluster aggregates synthesized at pH (a) 4.2 and (b) 5.9. Black and red traces correspond to the CD signals before and after the ultrasonication.

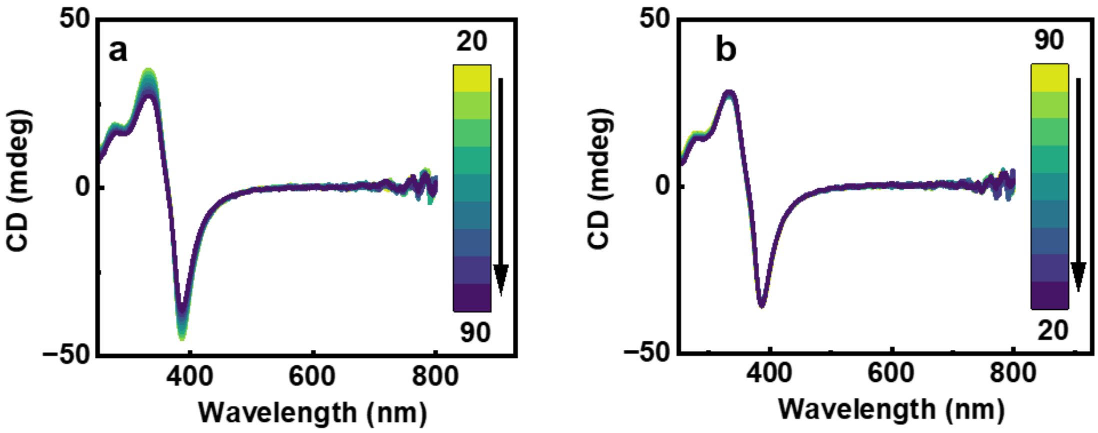

Figure S10. Temperature dependent CD of the Au cluster aggregates synthesized under pH 4.2 with (a) heating the system from 10 °C to 90 °C and (b) cooling back to room temperature.

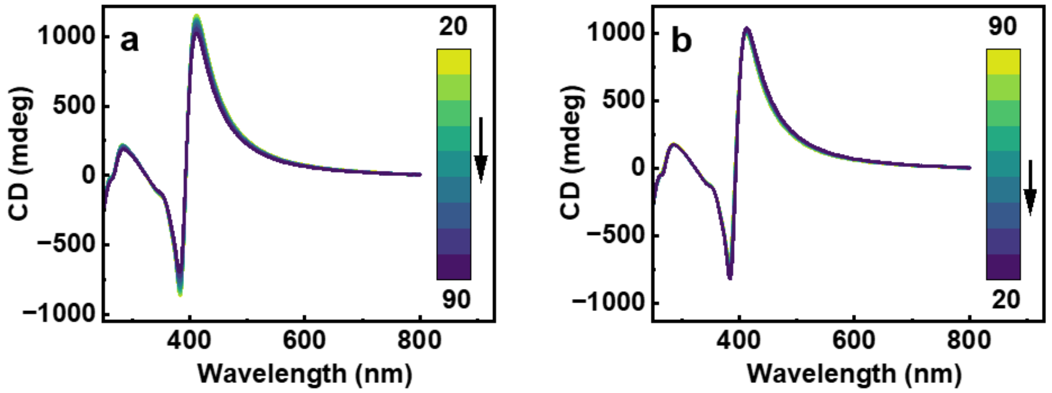

Figure S11. Temperature dependent CD of the Au cluster aggregates synthesized at pH 5.9 with (a) heating the system from 10 °C to 90 °C and (b) cooling back to room temperature.

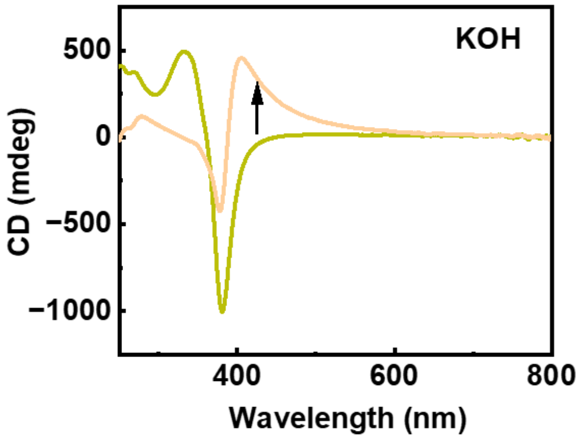

Figure S12. CD spectra of Au cluster aggregates synthesized in presence of L-cysteine and KOH as base.

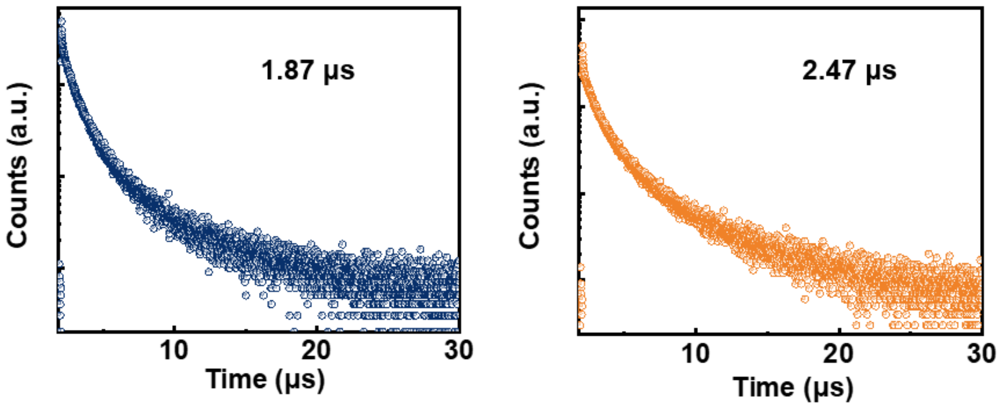

Figure S13. Lifetime decay plot of the citrate mediated Au clusters synthesized at pH 5.9 using L- (blue trace) and D- (orange trace) cysteine.

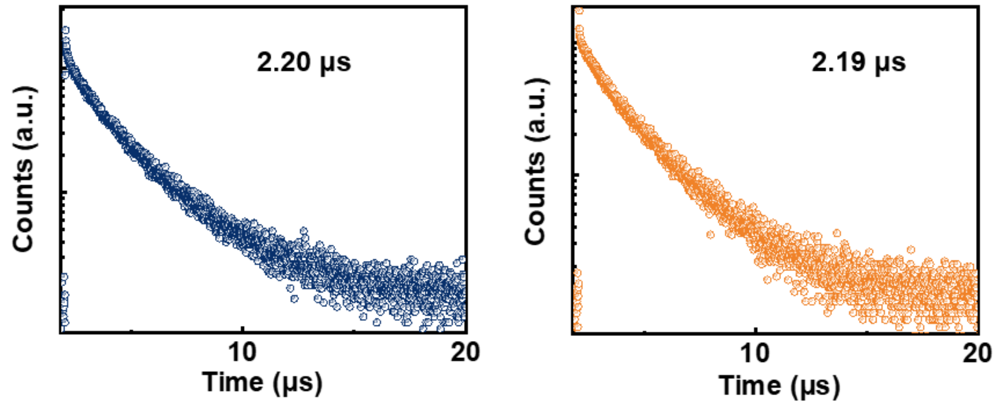

Figure S14. Lifetime decay plot of the citrate mediated Au clusters synthesized at pH 6.5 using L- (blue trace) and D- (orange trace) cysteine.

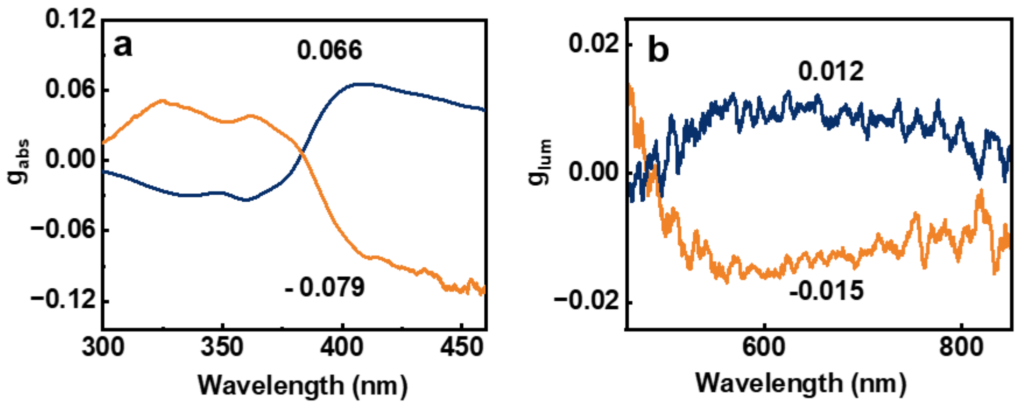

Figure S15. (a) g_{abs} and (b) g_{lum} plot of the citrate mediated Au clusters at pH 5.9. Blue and orange traces correspond to Au clusters synthesized using L- and D-cysteine, respectively.

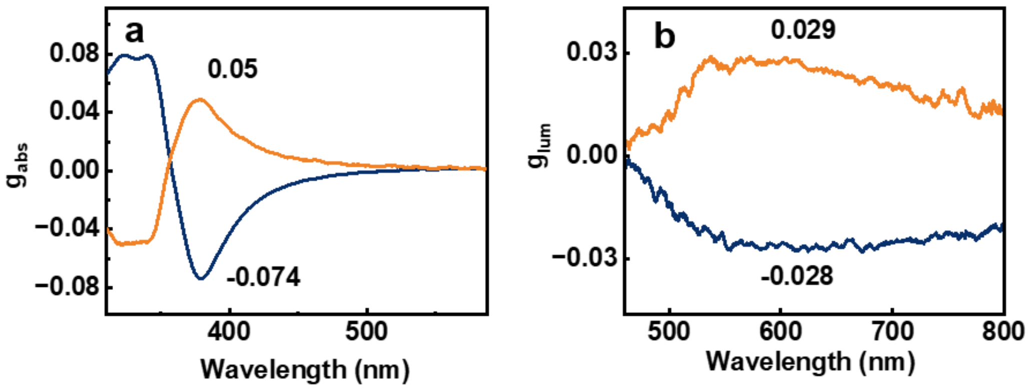

Figure S16. (a) g_{abs} and (b) g_{lum} plot of the citrate mediated Au clusters synthesized in presence of base (pH = 6.5). Blue and orange traces correspond to Au clusters synthesized using L- and D-cysteine,, respectively.

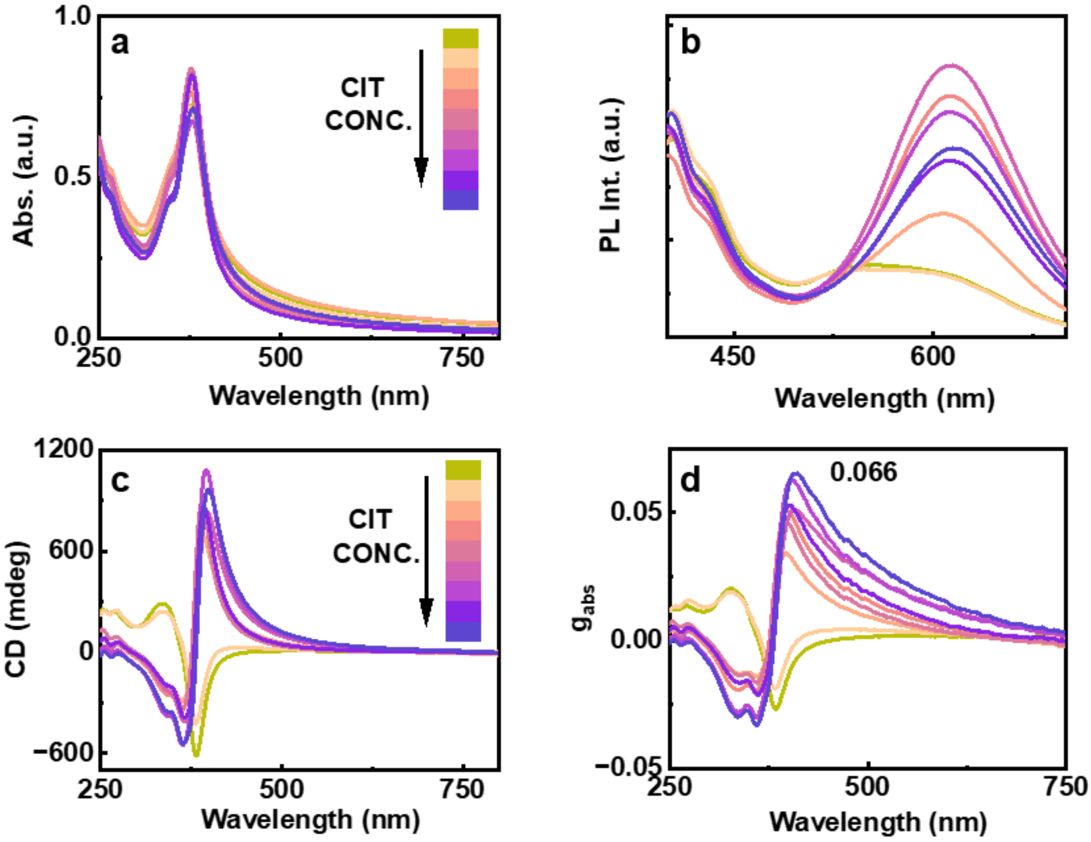

Figure S17. (a) Absorption, (b) PL emission, (c) CD and (d) g_{abs} plots of the L-cysteine stabilized Au clusters synthesized with different concentration of sodium citrate.

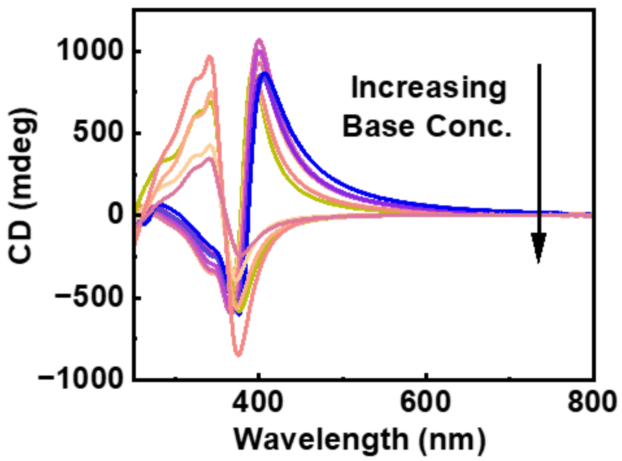

Figure S18. Optimization of base concentration for chiral inversion in L-cysteine stabilized Au clusters synthesized in presence of sodium citrate.

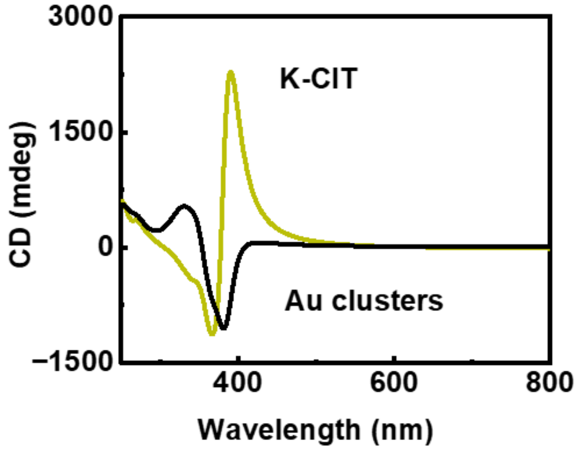

Figure S19. CD spectra of Au cluster aggregates synthesized in presence of L-cysteine and potassium citrate.

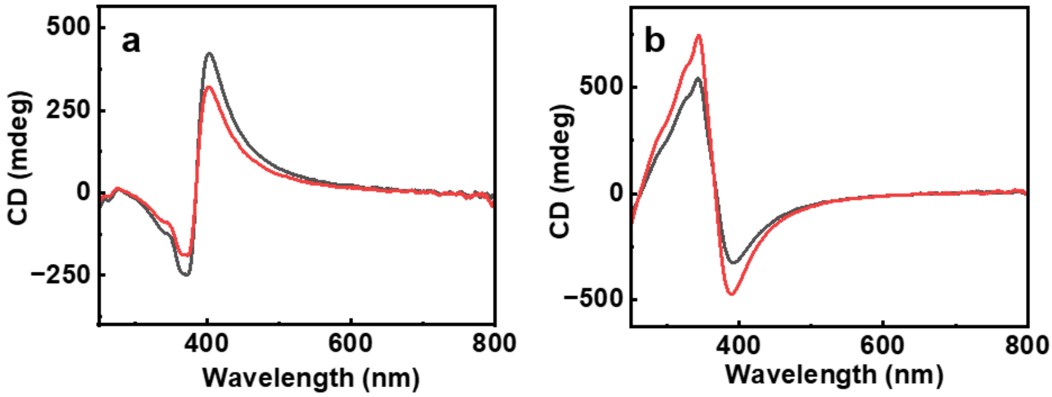

Figure S20: Effect of sonication on the citrate mediated Au clusters synthesized at pH (a) 5.9 and (b) 6.5. Black and red traces correspond to the CD signal before and after the ultrasonication.

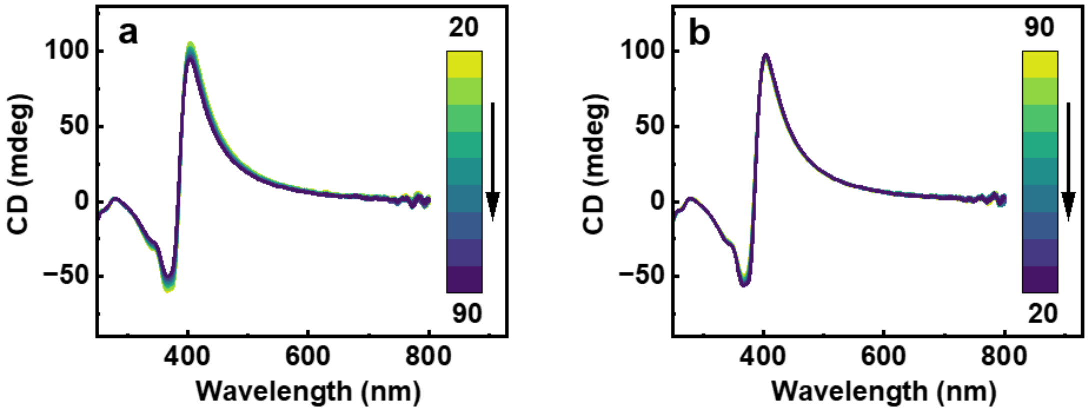

Figure S21. Temperature dependent CD of the citrate mediated Au clusters aggregates synthesized at 5.9 pH with (a) heating the system from 10 °C to 90 °C and (b) cooling back to room temperature.

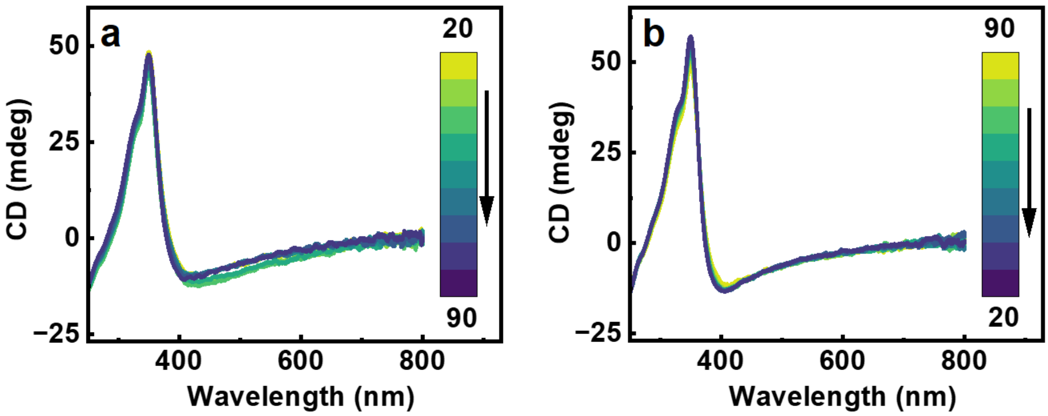

Figure S22. Temperature dependent CD of the citrate mediated Au clusters aggregates synthesized at 6.5 pH with (a) heating the system from 10 °C to 90 °C and (b) cooling back to room temperature.

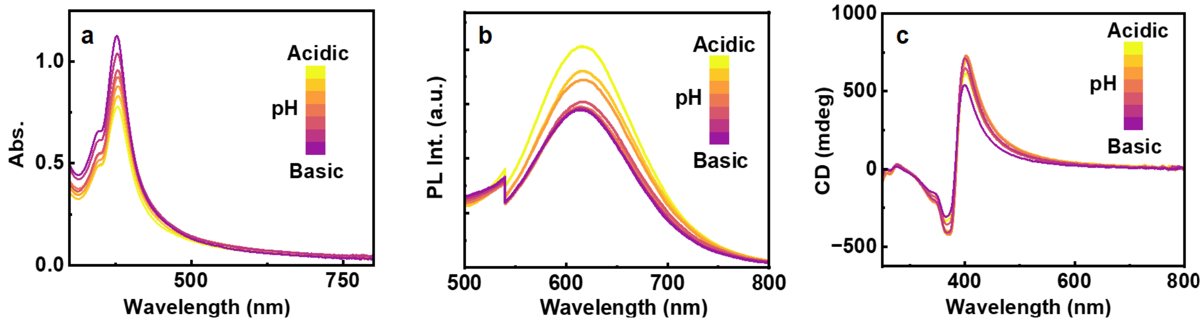

Figure S23. (a) Absorption, (b) emission, and (c) CD spectra of the clusters dissolved under different pH solutions.
